# Hemochromatosis, Iron Overload–Related Diseases, and Pancreatic Cancer Risk in the Surveillance, Epidemiology, and End Results (SEER)-Medicare

**DOI:** 10.1158/1055-9965.EPI-21-0476

**Published:** 2021-09-03

**Authors:** Sachelly Julián-Serrano, Fangcheng Yuan, Michael J. Barrett, Ruth M. Pfeiffer, Rachael Z. Stolzenberg-Solomon

**Affiliations:** 1 Division of Cancer Epidemiology and Genetics, NCI, NIH, Bethesda, Maryland.; 2 Information Management Services, Inc., Rockville, Maryland.

## Abstract

**Background:**

Experimental studies suggest that iron overload might increase pancreatic cancer risk. We evaluated whether prediagnostic hemochromatosis and iron-overload diseases, including sideroblastic and congenital dyserythropoietic anemias, and non–alcoholic-related chronic liver disease (NACLD) were associated with pancreatic cancer risk in older adults.

**Methods:**

We conducted a population-based, case–control study within the U.S. Surveillance, Epidemiology, and End Results Program (SEER)-Medicare linked data. Incident primary pancreatic cancer cases were adults > 66 years. Controls were alive at the time cases were diagnosed and matched to cases (4:1 ratio) by age, sex, and calendar year. Hemochromatosis, iron-overload anemias, and NACLD were reported 12 or more months before pancreatic cancer diagnosis or control selection using Medicare claims data. Adjusted unconditional logistic regression models were used to calculate ORs and 95% confidence intervals (CI) between hemochromatosis, sideroblastic and congenital dyserythropoietic anemias, NACLD, and pancreatic cancer.

**Results:**

Between 1992 and 2015, 80,074 pancreatic cancer cases and 320,296 controls were identified. Overall, we did not observe statistically significant associations between hemochromatosis, sideroblastic anemia, or congenital dyserythropoietic anemia and pancreatic cancer; however, sideroblastic anemia was associated with later primary pancreatic cancer (OR, 1.30; 95% CI, 1.03–1.64). NACLD was associated with first (OR, 1.10; 95% CI, 1.01–1.19), later (OR, 1.17; 95% CI, 1.02–1.35), and all (OR, 1.12; 95% CI, 1.04–1.20) pancreatic cancer.

**Conclusions:**

Overall hemochromatosis and iron-overload anemias were not associated with pancreatic cancer, whereas NACLD was associated with increased risk in this large study of older adults.

**Impact:**

These results partly support the hypothesis that iron-overload diseases increase pancreatic cancer risk.

## Introduction

Although pancreatic cancer (pancreatic cancer) only accounts for 3% of all incident cancers, it is highly fatal with a 5-year survival rate of 10% and ranks third for cancer mortality in the United States (1). Experimental studies of iron overload suggest that iron accumulation in pancreatic islets impairs insulin secretion and β-cell function and accelerates pancreatic β-cell death (2). Higher serum iron has also been found to be associated with pancreatic cancer, although not consistently (3–5). Previous epidemiologic studies have identified associations of red meat consumption and heme iron with type 2 diabetes mellitus and pancreatic cancer risk (6). Hemochromatosis is a disease characterized by iron overload, and patients with sideroblastic anemia, congenital dyserythropoietic anemia, and chronic liver disease (CLD) are prone to iron overload (7). We investigated the association of hemochromatosis, sideroblastic and congenital dyserythropoietic anemias, and non–alcoholic-related CLD (NACLD) with pancreatic cancer in a large U.S. population-based case–control study in older people. We hypothesize that iron overload–related diseases are associated with increased pancreatic cancer risk.

## Materials and Methods

We conducted a population-based, nested case–control study within the Surveillance Epidemiology, and Ends Results Program (SEER)-Medicare with details described elsewhere (8). Pancreatic cancer cases were defined as individuals with primary malignant disease [first and later (occurring after another cancer diagnosis other than pancreatic cancer) primaries; International Classification of Disease for Oncology, ICD-O codes C25.0-C25.9, malignant code 3], identified from the SEER-Medicare Patient Entitlement Diagnosis Summary File (PEDSF). Cases were diagnosed at ages 66 to 99 years, between 1992 and 2015. Individuals diagnosed with cancer by death certificate and at autopsy were excluded. To avoid surveillance bias (i.e., diagnosis of iron overload diseases led to increased surveillance for cancer), we excluded the 12-month period prior to case diagnosis from the ascertainment of iron-overload diseases. pancreatic cancer cases and controls were eligible they were if they had at least one Medicare claim (MEDPAR, NCH, or OUTPATIENT) more than 12 months prior to diagnosis.

A total of 320,296 population-based controls (4:1 ratio for each case) were randomly selected from a 5% subcohort of Medicare-enrolled beneficiaries. Controls were selected from the Summarized Denominator (SUMDENOM) file that contains demographic and claims data from a 5% random sample of Medicare recipients residing in SEER areas who never developed cancer and from a 5% sample of recipients in the PEDSF who developed cancer. Pancreatic cancer cases and matched controls were required to have a minimum of 13 months of Medicare Part A and B, health maintenance organization coverage prior to diagnosis/selection, making the minimum age at diagnosis 66 years. Controls were alive and either free of cancer or pancreatic cancer (for first primary or later primaries, respectively) as of July 1 of the calendar year that the case was diagnosed and frequency matched to the pancreatic cancer cases by age (66–69, 70–74, 75–79, 80–84, 85–89, 90–94, 95–99), sex, and calendar year of diagnosis.

We used the ICD-9 codes from the Medicare claim files to identify hemochromatosis (275.0, 275.01, and 275.03), sideroblastic (285.0), and congenital dyserythropoietic (285.8) anemias and NACLD (571.4, 571.41, 571.42, 571.49, 571.5, 571.6, 571.8, and 571.9). An individual was classified as having a specific condition if there were at least 1 inpatient claim or 2 physician/outpatient claims at least 30 days apart. We required the conditions to be present at least 12 months prior to case diagnosis or control selection to avoid surveillance bias and differential assessment of exposure status.

Demographic characteristics for cases and controls were compared using *t* tests for continuous variables and *χ*^2^ tests for categorical values. We used unconditional logistic regression models to calculate ORs and 95% confidence intervals (CI) for the association of iron overload–related diseases with first, later, and all primary pancreatic cancer, adjusting for the a *priori*–selected covariates that are known to be associated with pancreatic cancer listed in Table 1 and for SEER registries.

**Table 1.** Demographic characteristics of SEER-Medicare pancreatic cancer cases and frequency-matched controls 1992–2015^a^.

	Pancreatic cancer cases^b^			
	Controls	First primary	Later primaries	All primaries
	(*N* = 320,296)	(*N* = 61,081)	(*N* = 18,993)	(*N* = 80,074)
**Characteristics**	*n* (%)	*n* (%)	*n* (%)	*n* (%)
**Age at diagnosis or selection, years**				
66–69	42,388 (13.2)	8,590 (14.1)	2,007 (10.6)	10,597 (13.2)
70–74	71,148 (22.2)	13,979 (22.9)	3,808 (20.0)	17,787 (22.2)
75–79	75,692 (23.6)	14,253 (23.3)	4,670 (24.6)	18,923 (23.6)
80–84	66,048 (20.6)	12,140 (19.9)	4,372 (23.0)	16,512 (20.6)
85–89	43,496 (13.6)	7,991 (13.1)	2,883 (15.2)	10,874 (13.6)
90–94	17,524 (5.5)	3,357 (5.5)	1,024 (5.4)	4,381 (5.5)
95–99	4,000 (1.2)	771 (1.3)	229 (1.2)	1,000 (1.2)
**Sex**				
Men	144,484 (45.1)	26,277 (43.0)	9,844 (51.8)	36,121 (45.1)
Women	175,812 (54.9)	34,804 (57.0)	9,149 (48.2)	43,953 (54.9)
**Race/ethnicity**				
Non-Hispanic white	264,161 (82.5)	49,619 (81.2)	16,171 (85.1)	65,790 (82.2)
Non-Hispanic black	23,841 (7.4)	6,179 (10.1)	1,705 (9.0)	7,884 (9.8)
Non-Hispanic Asian	14,483 (4.5)	2,176 (3.6)	442 (2.3)	2,618 (3.3)
Hispanic	7,893 (2.5)	1,227 (2.0)	237 (1.2)	1,464 (1.8)
Other/missing	9,918 (3.1)	1,880 (3.1)	438 (2.3)	2,318 (2.9)
**Calendar year of diagnosis or selection**				
1992–2000	61,352 (19.2)	12,382 (20.3)	2,956 (15.6)	15,338 (19.2)
2001–2005	73,460 (22.9)	14,279 (23.4)	4,086 (21.5)	18,365 (22.9)
2006–2009	71,592 (22.4)	13,537 (22.2)	4,361 (23.0)	17,898 (22.4)
2010–2015	113,892 (35.6)	20,883 (34.2)	7,590 (40.0)	28,473 (35.6)
**Months of coverage, median (IQR)**				
Part A/B/non-HMO	67 (42–82)	71 (42–84)	74 (48–85)	72 (43–84)
Part D	18 (0–52)	17 (0–48)	15 (0–49)	16 (0–49)
**Average physician visits/6 months, median (IQR)**	3.0 (1.3–5.5)	3.1 (1.4–5.8)	4.3 (2.3–7.1)	3.4 (1.5–6.1)
**Low-income subsidy/Medicaid eligibility**				
Ever	37,034 (11.6)	6,695 (11.0)	1,720 (9.1)	8,415 (10.5)
Never	130,922 (40.9)	24,398 (39.9)	9,176 (48.3)	33,574 (41.9)
Unknown	152,340 (47.6)	29,988 (49.1)	8,097 (42.6)	38,085 (47.6)
**Co-morbidities diagnoses**				
Overweight or obesity	17,980 (5.6)	4,113 (6.7)	1,398 (7.4)	5,511 (6.9)
Smoking behavior-related^c^	23,803 (7.4)	5,787 (9.5)	2,472 (13.0)	8,259 (10.3)
Chronic obstructive pulmonary disease	59,294 (18.5)	12,734 (20.8)	4,705 (24.8)	17,439 (21.8)
Alcohol-related^d^	14,294 (4.5)	4,102 (6.7)	1,475 (7.8)	5,577 (7.0)
Acute or chronic pancreatitis	3,609 (1.1)	1,685 (2.8)	591 (3.1)	2,276 (2.8)
Type 2 diabetes mellitus	90,232 (28.2)	21,993 (36.0)	7,149 (37.6)	29,142 (36.4)
**Hemochromatosis**	653 (0.2)	137 (0.2)	49 (0.3)	186 (0.2)
**Sideroblastic anemia**	993 (0.3)	192 (0.3)	108 (0.6)	300 (0.4)
**Congenital dyserythropoietic anemia**	1,375 (0.4)	262 (0.4)	136 (0.7)	398 (0.5)
**NACLD**	3,316 (1.0)	791 (1.3)	311 (1.6)	1,102 (1.4)
Liver cirrhosis	1,113 (0.3)	279 (0.5)	105 (0.6)	384 (0.5)
Fatty liver disease	1,503 (0.5)	359 (0.6)	146 (0.8)	505 (0.6)

Abbreviations: HMO, health maintenance organizations; IQR, interquartile range; NACLD, non–alcoholic-related chronic liver disease; SEER, Surveillance, Epidemiology, and Ends Results Program.

^a^Controls were frequency matched to the pancreatic cancer cases by age category (66–69. 70–74, 75–79, 80–84, 85–89, 90–94, and 95–99), sex, and calendar year of diagnosis. International Classification of Diseases – 9th edition included: hemochromatosis (275.0, 275.01, and 275.03), sideroblastic (285.0), and congenital dyserythropoietic (285.8) anemias and NACLD (571.4, 571.41, 571.42, 571.49, 571.5, 571.6, 571.8, and 571.9).

^b^First primary pancreatic cancer was defined as pancreatic cancer occurring as a first malignancy and later primaries pancreatic cancer were defined as primary pancreatic cancer occurring as second or third malignancy between 1992 and 2015.

^c^Any personal history of tobacco use or nondependent tobacco use disorder.

^d^Any diagnosis of alcohol-induced liver disorders, alcohol-induced psych/neurologic disorders, alcohol intoxication, nondependent alcohol abuse, or personal history of alcoholism.

## Results

In total, 80,074 (61,081 first and 18,993 later primaries) incident pancreatic cancer cases were identified. Compared with controls, cases were more often non-Hispanic black and overweight/obese, and had chronic obstructive pulmonary disease, smoking, alcohol, pancreatitis, and type 2 diabetes–related diagnoses (Table 1). Hemochromatosis or iron-overload anemias were not associated with pancreatic cancer (Table 2). We observed a significant 30% increased risk for later primary pancreatic cancer among patients diagnosed with sideroblastic anemia. NACLD was significantly associated a 10%–17% elevated risk for first, later, and all pancreatic cancer (Table 2).

**Table 2.** Associations between hemochromatosis, iron-overload anemias, and NACLD with pancreatic cancer in the SEER-Medicare 1992–2015^a^.

	First primary pancreatic cancer	Later primaries pancreatic cancer	All primaries pancreatic cancer
Conditions	(*N* = 61,081)	(*N* = 18,993)	(*N* = 80,074)
**Hemochromatosis**			
** **Model 1 OR (95% CI)^b^	1.08 (0.89–1.31)	1.06 (0.76–1.46)	1.07 (0.91–1.26)
** **Model 2 OR (95% CI)^c^	1.04 (0.86–1.26)	1.05 (0.76–1.46)	1.04 (0.88–1.23)
**Sideroblastic anemia**			
** **Model 1 OR (95% CI)^b^	1.00 (0.85–1.18)	1.33 (1.06–1.68)	1.10 (0.97–1.26)
** **Model 2 OR (95% CI)^c^	0.93 (0.79–1.09)	1.30 (1.03–1.64)	1.04 (0.91–1.18)
**Congenital dyserythropoietic anemia**			
** **Model 1 OR (95% CI)^b^	0.99 (0.86–1.13)	1.16 (0.95–1.42)	1.04 (0.93–1.17)
** **Model 2 OR (95% CI)^c^	0.95 (0.83–1.10)	1.13 (0.92–1.38)	1.01 (0.90–1.13)
**NACLD**			
** **Model 1 OR (95% CI)^b^	1.21 (1.12–1.32)	1.27 (1.11–1.46)	1.23 (1.15–1.32)
** **Model 2 OR (95% CI)^c^	1.10 (1.01–1.19)	1.17 (1.02–1.35)	1.12 (1.04–1.20)
** **Liver cirrhosis			
** **Model 1 OR (95% CI)^b^	1.20 (1.04–1.38)	1.20 (0.95–1.51)	1.20 (1.07–1.35)
** **Model 2 OR (95% CI)^c^	1.08 (0.94–1.25)	1.10 (0.87–1.40)	1.09 (0.97–1.23)
** **Fatty liver disease			
** **Model 1 OR (95% CI)^b^	1.22 (1.08–1.38)	1.34 (1.10–1.64)	1.26 (1.13–1.39)
** **Model 2 OR (95% CI)^c^	1.10 (0.97–1.24)	1.24 (1.02–1.52)	1.14 (1.02–1.26)

Abbreviation: NACLD, non–alcoholic-related chronic liver disease.

^a^First primary pancreatic cancer was defined as pancreatic cancer occurring as a first malignancy and later primaries pancreatic cancer were defined as primary pancreatic cancer occurring as second or third malignancy between 1992 and 2015. International Classification of Diseases – 9th edition included: hemochromatosis (275.0, 275.01, and 275.03), sideroblastic (ICD-9 285.0), and congenital dyserythropoietic (ICD-9 285.8) anemias and NACLD (ICD-9 571.4, 571.41, 571.42, 571.49, 571.5, 571.6, 571.8, and 571.9).

^b^Adjusted for age category (66–69, 70–74, 75–79, 80–84, 85–89, 90–94, and 95–99 years); sex (men vs. women); calendar year of selection (1992–2000, 2001–2005, 2006–2009, 2010–2015); race/ethnicity (non-Hispanic white, non-Hispanic black, Asian/Pacific Islander, Hispanics, and other/unknown); SEER registry; average number of physician visits (quintiles); Medicaid/low-income subsidy (ever, never, unknown); average duration of Medicare Part A, B, or non-HMO coverage (quintiles); overweight/obesity (yes vs. no); smoking behavior-related diagnosis (yes vs. no); chronic obstructive pulmonary disease (yes vs. no); alcohol-related diagnosis (yes vs. no); and CLD (yes vs. no).

^c^Additionally adjusted for type 2 diabetes mellitus (yes vs. no) and pancreatitis (yes vs. no).

## Discussion

Overall, we did not observe associations between diagnosed hemochromatosis, iron-overload anemias, and pancreatic cancer in this large, nested case-control study within the SEER-Medicare population. We observed significant elevated pancreatic cancer risks for NACLD, particularly for nonalcoholic fatty liver disease (NAFLD) and cirrhosis (NAC). As adiposity and diabetes are risk factors for NAFLD, NAC, and pancreatic cancer, residual confounding is likely present because we use claims data for adjustment. We speculate that the higher later primary pancreatic cancer risk among participants with sideroblastic anemia might reflect effects of therapy for the earlier cancer(s). Limitations of our study include the low proportion of participants with hemochromatosis or iron-overload anemias diagnoses, incomplete ascertainment of the conditions, and the older age of our study population. Our results partly support the hypothesis that diagnosed iron-overload diseases are associated with pancreatic cancer in older people.

## Authors' Disclosures

M.J. Barrett reports other support from NCI during the conduct of the study. No disclosures were reported by the other authors.
